# Ultrasonography of the distal limbs in Nellore and Girolando calves 8 to 12 months of age

**DOI:** 10.1186/1746-6148-10-102

**Published:** 2014-04-28

**Authors:** Pryscilla VR Gonçalves, Luiz AF Silva, Luiz H Silva, Ana Paula A Costa, Nathalia Bragato, Julio R Cardoso, Johann Kofler, Naida C Borges

**Affiliations:** 1 Department of Veterinary Medicine, Veterinary and Animal Science School, Federal University of Goias, Campus Samambaia, Caixa Postal 131, Goiânia, Goias State CEP 74001-970, Brazil; 2 Department of Animal Anatomy, Biologycal Sciences Institute, Federal University of Goias, Campus Samambaia, Caixa Postal 131, Goiânia, Goias State CEP 74001-970, Brazil; 3 Department of Farm Animals and Veterinary Public Health, Clinic for Ruminants, University of Veterinary Medicine Vienna, A-1210 Vienna, Austria

**Keywords:** Cattle, Radiography, Ultrasonography, Tendons, Joints, Limbs, Digits

## Abstract

**Background:**

Ultrasonography can be used anywhere and allows rapid, noninvasive differentiation of soft tissue structures of the musculoskeletal system. The objectives of this study were to describe the ultrasonographic appearance of the structures of the metacarpo-/metatarsophalangeal and the interphalangeal joints, the appearance of the growth plates of the distal metacarpus/metatarsus and of the proximal phalanx and to measure the cross-sectional dimensions of the DDFT and SDFT in Nellore and Girolando calves eight to 12 months of age.

**Results:**

In the longitudinal dorsal view the common digital extensor tendon and the digital extensor tendon were depicted as echogenic parallel fiber bundles located directly under the skin. The joint spaces appeared as anechoic interruptions of the hyperechogenic bone surfaces. The normal amount of synovial fluid could not be depicted. The growth plates were seen as anechoic interruptions of the bone surface proximal and distal to the fetlock joint space. In transverse sonograms of the distal palmar/plantar regions, the flexor tendons and branchs of the suspensory ligament were imaged as echogenic structures. The lumen of the digital flexor tendon sheath could not be imaged in these normal cattle. The thin digital distal annular ligament and the reversal of positions of the DDFT and SDFT could be appreciated. No significant differences were found between the cross-sectional measurements of the DDFT and the SDFT from Nellore and Girolando in any age, thoracic/pelvic limbs, right/left sides and lateral/medial digits.

**Conclusions:**

The results of this study establish important ultrasonographic reference data of the normal structures of the distal limbs and the normal dimensions of the flexor tendons in Nellore and Girolando calves for use in clinical practice.

## Background

Previously, high prevalences of claw disorders and lameness of 25% to 50.2% have been reported in heifers [1-5]. This situation must be considered as critical from an economical point of view, becauce these heifers are intended to be future milk or meat producing cattle [6].

Diseases of the distal limbs are very common and occur in cattle of all ages and breeds; they can be divided into infectious and non-infectious disorders [7-10]. Among non-infectious causes of lameness, osteochondrosis is more frequent in fast growing bovines on a high energy diet [10-13]. Metaphyseal and subchondral osteomyelitis has been reported frequently in calves [14] with less than three months of age (44.4%), evenly distributed between three and 12 months of age (6.3-10.4%) and even found in animals over 12 months of age (31%) [15].

In lame cattle the diagnosis starts with an accurate clinical exam followed by ultrasonography if necessary [16,17] and/or synovial fluid analysis [9,18]. Additional diagnostic imaging techniques can be applied such as radiography and arthroscopy [15,19,20].

Ultrasonography is an imaging modality that can be used anywhere and allows rapid, noninvasive differentiation of soft tissue structures of the bovine musculoskeletal system [17,21]. Ultrasonography of the tendons and ligaments in the metacarpal/metatarsal region is one of the easiest methods to study the echogenicity and the cross-sectional diameters of the superficial digital flexor tendon (SDFT), the deep digital flexor tendon (DDFT) and the suspensory ligament (SL) in different breeds of horses [22] and cattle [23].

Considering that infectious and non-infectious disorders of the distal limbs occur frequently and that ultrasonography would be a great option for evaluation of the soft tissues, joints and bone surfaces in this region, the purpose of this study was to map the distal limb region in healthy Nellore and Girolando calves in order to obtain ultrasonographic references in these breeds for the interpretation of potentially pathological findings. The objectives of this study were to describe the ultrasonographic appearance of the soft tissue structures of the metacarpo-/metatarso-phalangeal and the interphalangeal joints, the appearance of the growth plates of the distal metacarpus/metatarsus, and to measure the cross-sectional dimensions of the DDFT and SDFT in these breeds.

## Methods

### Animals

Sixteen healthy calves (Nellore, n = 9; Girolando, n = 7) were examined by ultrasonography in three different periods of growth at the age of eight, 10 and 12 months.

The animals were kept in covered sheds with concrete floors, received water *ad libitum* and were fed daily with corn silage supplemented with concentrate corresponding to 2% of body weight in dry matter (Ration Performance 18 AE, Boiforte Agricultural Products LTDA, Goiânia, Brazil).

The Ethics Committee of Federal University of Goiás (009/2008) approved all procedures.

### Exclusion of lameness

A lameness evaluation, consisting of locomotion scoring while standing and walking was performed in every animal daily and inspection and palpation of the distal region was carried out [17]. Lame animals were excluded from the study.

Radiographs of the distal limbs were obtained in dorsopalmar/dorsoplantar and lateromedial projections, the central ray was positioned at the joint space of the fetlock joint , according to Bargai et al. [19].

### Ultrasonographic examination

Ultrasonography was performed in lateral recumbency with calves sedated with 0.01 mg/Kg xylazine 2%, intravenously (Calmium®, Agener, São Paulo, Brazil).

The region around the metacarpophalangeal and the metatarsophalangeal joint down to the coronary band of the hooves was clipped, cleaned with water, and acoustic coupling gel was applied. For all calves, a 10.0 MHz linear probe without a stand-off pad was used (My Lab™ 30 Vet®, Esaote Group, Genova, Italy).

**Figure 1:**
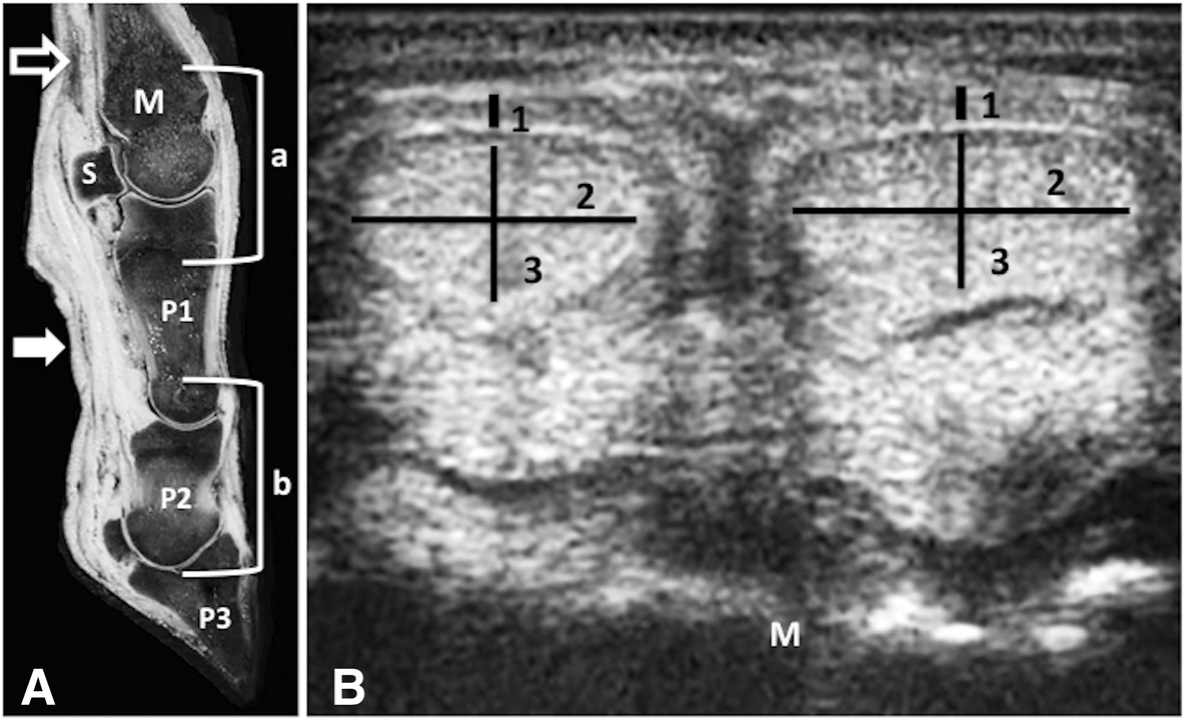
**(A) Longitudinal anatomic section of a frozen specimen illustrating the transducer positions: dorsal longitudinal view of the metacarpophalangeal joint (a) and the interphalangeal joints (b); palmar/plantar transverse view for the evaluation of tendons and ligaments of the distal metacarpal/metatarsal region (arrow with white frame) and the pastern region (white arrow). (B)** The transverse sonogram of a 12 month old Girolando calf (10.0 MHz linear transducer) shows how the measurements were carried out to assess the thickness of the superficial digital flexor tendon (SDFT) (1), and the thickness (2) and the width (3) of the deep digital flexor tendon (DDFT). M-metacarpal metaphysis, S-proximal sesamoidal bone, P1-first phalanx, P2-second phalanx, P3-third phalanx.

The probe was positioned at the dorsal aspect in a longitudinal plane (dorsal view) for the examination of the extensor tendon and the bone surface including the joint space of the metacarpo-/metatarsophalangeal joint (Figure 1). For the palmar/plantar views the probe was positioned in the transverse plane three centimeters proximal to the apex of the proximal sesamoidal bones in order to image the digital flexor tendons, the SL and the branch of the SL to the SDFT. In addition, a second palmar/plantar plane was used with positioning of the probe in the pastern region immediately distal of the dew claws (Figure 1). The digital joints of both hindlimbs and forelimbs were evaluated. All ultrasonographic examinations and measurements were performed by the first author (PVRG) according to the literature [17,21].

### Evaluated structures

The following anatomical structures were examined and the indicated parameters were evaluated:

(1) Distal metacarpal and metatarsal condyles: echogenicity and characteristics of the bone surface, cartilaginous physis, joint space and the joint cartilage of the fetlock joint;

(2) Proximal and middle phalanx: echogenicity and characteristics of the bone surface, cartilaginous physis of the proximal phalanx, joint space of the proximal interphalangeal joint and the joint cartilage;

(3) Distal phalanx: echogenicity and characteristics of the extensor process, joint space of the distal interphalangeal joint;

(4) Soft tissues: echogenicity and structure of the tendons (common digital extensor tendon, extensor tendon of third digits, SDFT, DDFT), ligaments (SL, branch of the SL to the SDFT, distal annular ligament), vessels (common digital palmar artery), joint pouches, joint capsule and digital flexor tendon sheath in dorsal and palmar/plantar views respectively.

Three parameters were measured in the digital flexors tendons: the dorso-palmar/plantar dimension (thickness) of the SDFT and cross-sectional diameters (thickness and width) (Figure 1) of the DDFT.

These examinations were performed as previously described in the literature [22-24].

### Statistical analysis

Statistical analyses were carried out using the Biostat program version 5.3. Three measurements of the same animal were aggregated to a single value. For all measurements, a mean and standard deviation were calculated. The assumption of normal distribution was proved using the Kolmogorov–Smirnov test. To test the differences between body weight, breeds, and ages in all views a T test was applied adopting significance at P ≤ 0.05.

## Results

One hundred and ninety-two (96 thoracic and 96 pelvic) distal limbs in 16 calves, at three different ages, were examined ultrasonographically. The mean body weight at the age of eight months was 139 ± 17 kg in Nellore and 133 ± 37 kg in Girolando (P = 0.101), at 10 months 209 ± 27 kg in Nellore and 215 ± 34 kg in Girolando (P = 0.433) and at 12 months 235 ± 34 kg in Nellore and 245 ± 36 kg in Girolando (P = 0.640). The absence of lesions in hoofs, tendons and bones was confirmed by clinical and radiographic examinations.

**Figure 2:**
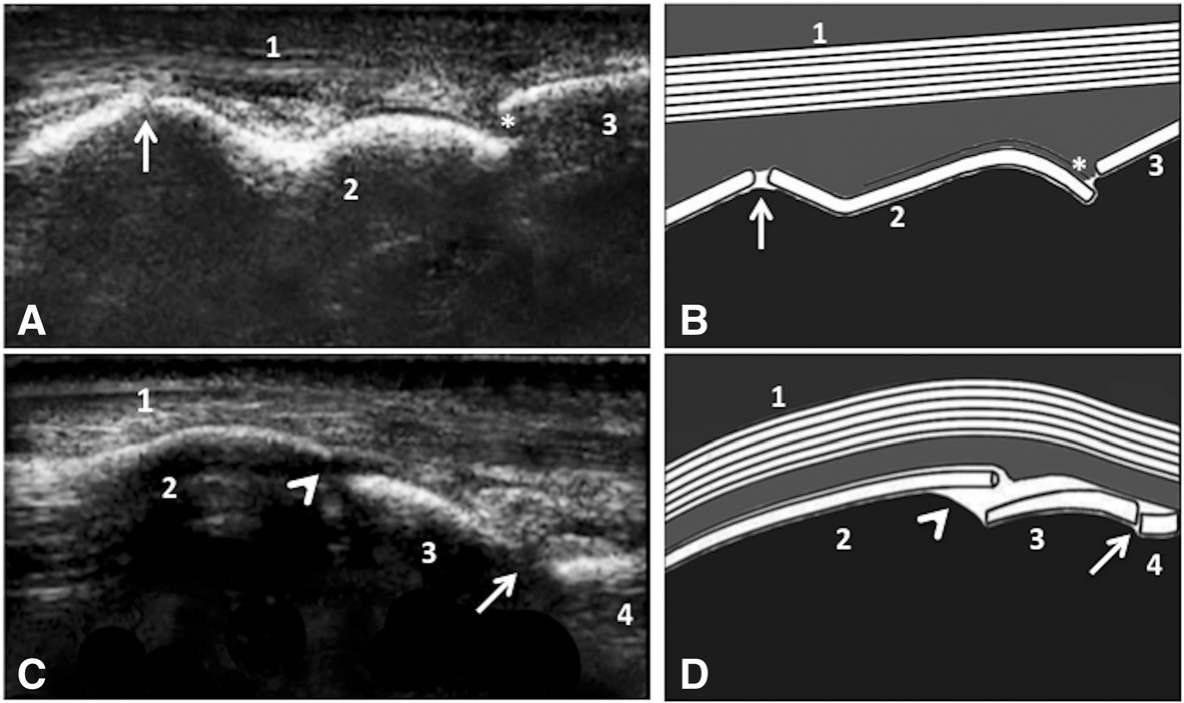
Dorsal longitudinal sonogram (10 MHz) of the metacarpophalangeal joint of an eight month old Nellore calf (A) and the interphalangeal joints of a six month old Girolando calf (C) with schematic drawings (B and D): in (A) and (B): 1-common digital extensor tendon, 2-metacarpal condyle, 3-proximal phalanx, anechoic interruption of the hyperechogenic bone surfaces representing the joint space (asterisk), normal growth plate of the distal metacarpus (arrow); in (C) and (D): 1- digital extensor tendon, 2-hyperechogenic contour of the proximal phalanx, 3-hyperechogenic contour of the second phalanx, 4-hyperechogenic contour of the distal phalanx, anechoic interruptions of the hyperechogenic bone surfaces representing the proximal interphalangeal (head arrow) and the distal interphalangeal joint (arrow) spaces.

In the dorsal view of the metacarpo-/metatarsophalangeal joint the common digital extensor tendon was depicted as an echogenic parallel fiber bundle (Figure 2) located directly under the skin. The bone surfaces of the metacarpal/metatarsal condyles and proximal, medial and distal phalanx appeared as hyperechogenic smooth contours (Figure 2).

The joint spaces of the metacarpo-/metatarsophalangeal joint and the proximal distal interphalangeal joints appeared as anechoic interruptions of the hyperechogenic bone surfaces (Figure 2). The joint capsules of the fetlock and the two interphalangeal joints appeared as echogenic structures adjoining closely at the articular surfaces. The normal amount of synovial fluid could not be depicted, therefore the joint pouches could not be outlined (Figure 2) in these normal cattle.

The growth plates of the distal metacarpal and metatarsal bones (Figure 2) and the proximal growth plate of the proximal phalanx were imaged in the three exams in all calves as very small, anechoic interruptions of the bone surface proximal and distal to the fetlock joint space, respectively.

**Figure 3:**
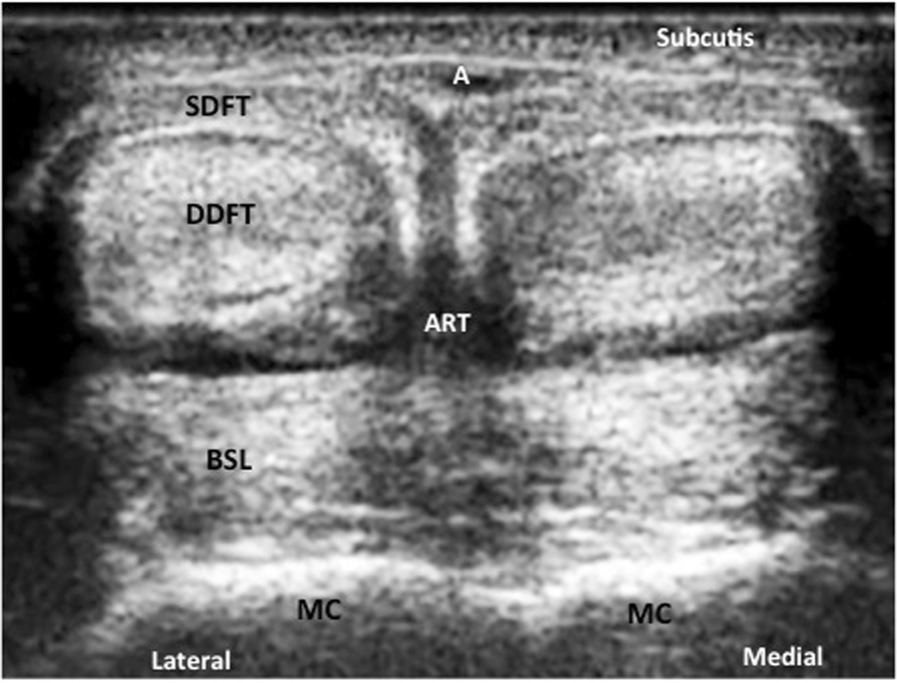
Transverse view of the distal palmar metacarpal region (10.0 MHz) region of an eight month old Nellore calf showing the normal appearance of superficial digital flexor tendon (SDFT); deep digital flexor tendon (DDFT); branch of the suspensory ligament to the SDFT (BSL-S); branches of the suspensory ligament (BSL); surface of the metacarpus (MC); common digital palmar artery (A); edge shadowing artifact (ART).

In transverse sonograms of the distal palmar/plantar metacarpal/metatarsal region the SDFT, DDFT, the branch of the SL to the SDFT and the branches of the SL were imaged as echogenic structures (Figure 3). The palmar/plantar bone surfaces of the metacarpal/metatarsal condyle appeared as hyperechogenic contours (Figure 3). In this study, in calves of all ages the SDFT was imaged as a half-moon shaped structure with a slight hypoechoic or echoic appearance. The same echogenicity was determined for the DDFT, the SL and the branch of the SL to the SDFT. The lumen of the digital flexor tendon sheath could not be imaged in these normal cattle. Both lateral and medial branches of the SL were imaged as echogenic, peanut-shaped masses each; the abaxial and the axial branch were hard to differentiate (Figure 3).

**Figure 4:**
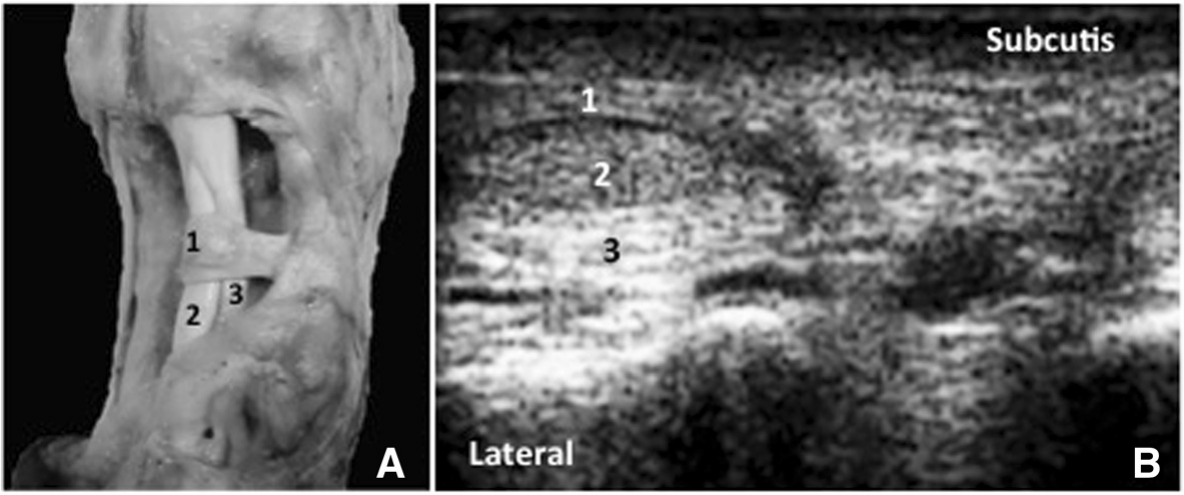
Comparison of the anatomic dissection of a specimen (A) with the sonoanatomy of the transverse palmar view (10.0 MHz) of the pastern region in a six month old Girolando calf (B) showing, 1-digital distal annular ligament, 2-deep digital flexor tendon, 3-superficial digital flexor tendon.

In the transverse sonograms of palmar/plantar pastern region the thin digital distal annular ligament and the reversed position of the DDFT and SDFT (Figure 4) were differentiated. The palmar/plantar bone surface of the proximal phalanx appeared as a hyperechogenic line (Figure 4). These ultrasonographically depicted structures were confirmed by gross anatomy (Figure 4).

All the mentioned soft tissue structures, joint spaces, bone contours and growth plates could be imaged in all the three repeated ultrasonographic examinations in all calves.

The measurements of the cross sectional diameters of the SDFT and the DDFT are shown in Table 1. No significant differences (P ≥ 0.05) were found when comparing the measurements from Nellore and Girolando in any age, thoracic/pelvic limbs, right/left sides and lateral/medial digits. A tendency of the dimensions of the SDFT and DDFT to increase over time was observed in Nellore and Girolando calves during the study period of four months (Table 1).

**Table 1:** Means and standard deviations (±SD) of the superficial digital flexor tendon (SDFT) and deep digital flexor tendon (DDFT) measures in millimeters (mm) of the Nellore and Girolando calves at three different ages

**Limb**	**Side**	**Digit**	**Nellore**			**Girolando**		
			**Tickness (mm)**		**Width (mm)**	**Tickness (mm)**		**Width (mm)**
			**SDFT**	**DDFT**	**DDFT**	**SDFT**	**DDFT**	**DDFT**
**8 months of age**								
**T**	**R**	**Lat**	1.98 ± 0.24	5.62 ± 0.74	12.98 ± 1.18	2.01 ± 0.19	5.23 ± 0.78	12.70 ± 0.55
		**Med**	1.98 ± 0.20	5.41 ± 0.70	13.49 ± 1.10	2.03 ± 0.19	4.70 ± 0.43	12.94 ± 1.10
	**L**	**Lat**	2.01 ± 0.19	5.53 ± 0.76	13.67 ± 0.99	1.97 ± 0.20	5.17 ± 0.68	12.57 ± 0.93
		**Med**	2.02 ± 0.20	5.38 ± 0.68	14.11 ± 1.07	1.94 ± 0.18	6.07 ± 0.50	12.90 ± 1.11
**P**	**R**	**Lat**	2.26 ± 0.19	5.79 ± 0.80	13.60 ± 1.01	2.04 ± 0.19	5.34 ± 0.64	12.66 ± 0.90
		**Med**	2.17 ± 0.18	5.78 ± 0.63	13.67 ± 0.96	1.90 ± 0.13	5.47 ± 0.51	12.84 ± 0.98
	**L**	**Lat**	2.11 ± 0.16	5.99 ± 0.86	13.24 ± 1.04	2.09 ± 0.17	5.63 ± 0.65	11.77 ± 1.43
		**Med**	2.06 ± 0.21	6.00 ± 0.69	13.78 ± 1.18	1.98 ± 0.18	5.51 ± 0.67	12.88 ± 0.99
**10 months of age**								
**T**	**R**	**Lat**	2.28 ± 0.21	6.44 ± 0.48	14.40 ± 0.60	2.29 ± 0.20	6.28 ± 0.64	14.27 ± 1.05
		**Med**	2.24 ± 0.11	6.27 ± 0.51	14.72 ± 0.66	2.36 ± 0.13	6.31 ± 0.60	14.34 ± 0.79
	**L**	**Lat**	2.29 ± 0.19	6.39 ± 0.48	14.78 ± 0.57	2.16 ± 0.16	6.30 ± 0.39	14.48 ± 0.58
		**Med**	2.23 ± 0.17	6.44 ± 0.42	14.73 ± 0.64	2.21 ± 0.14	6.47 ± 0.32	14.64 ± 0.74
**P**	**R**	**Lat**	2.53 ± 0.20	6.45 ± 0.49	14.75 ± 0.55	2.31 ± 0.16	6.56 ± 0.61	14.73 ± 0.61
		**Med**	2.44 ± 0.13	6.63 ± 0.38	14.68 ± 0.62	2.33 ± 0.15	6.65 ± 0.61	14.46 ± 0.69
	**L**	**Lat**	2.37 ± 0.17	6.68 ± 0.49	14.71 ± 0.58	2.37 ± 0.18	6.76 ± 0.20	14.88 ± 0.60
		**Med**	2.37 ± 0.21	6.96 ± 0.36	14.71 ± 0.65	2.33 ± 0.18	6.57 ± 0.26	14.13 ± 0.76
**12 months of age**								
**T**	**R**	**Lat**	2.50 ± 0.21	6.68 ± 0.48	15.08 ± 0.43	2.46 ± 0.23	6.56 ± 0.43	14.78 ± 0.74
		**Med**	2.50 ± 0.24	6.52 ± 0.49	15.38 ± 0.66	2.53 ± 0.14	6.61 ± 0.36	14.96 ± 0.94
	**L**	**Lat**	2.63 ± 0.20	6.75 ± 0.40	15.16 ± 0.35	2.40 ± 0.16	6.46 ± 0.31	15.24 ± 0.63
		**Med**	2.46 ± 0.15	6.60 ± 0.40	15.30 ± 0.55	2.49 ± 0.12	6.62 ± 0.30	15.28 ± 0.56
**P**	**R**	**Lat**	2.68 ± 0.20	7.00 ± 0.43	16.49 ± 0.42	2.51 ± 0.20	6.91 ± 0.27	15.28 ± 0.59
		**Med**	2.64 ± 0.15	6.88 ± 0.37	15.50 ± 0.57	2.53 ± 0.14	7.00 ± 0.37	15.04 ± 0.66
	**L**	**Lat**	2.61 ± 0.23	7.07 ± 0.36	15.33 ± 0.40	2.54 ± 0.14	6.93 ± 0.18	15.46 ± 0.46
		**Med**	2.66 ± 0.13	7.21 ± 0.31	15.52 ± 0.50	2.57 ± 0.12	6.80 ± 0.32	14.70 ± 0.50

T test adopting P ≤ 0.05.

T = thoracic limbs; P = pelvic limbs; R = right side; L = left side; Lat = lateral; Med = medial.

## Discussion

The normal ultrasonographic appearances of the soft tissue and bone surfaces of bovine digits in adult cattle [23-26], of the carpal joint [27,28], tarsal joint [29], femoropatellar joint [17,30], shoulder joint [31] and coxofemoral joint [17,32,33] have been previously reported in adult ruminants and calves of European and North American breeds. However, there is only one report describing the ultrasonographic appearance of the distal limbs in adult Nellore cattle [34].

In the present study, the normal ultrasonographic appearance of the metacarpo-/metatarsophalangeal and the interphalangeal joints in Nellore and Girolando cattle from eight to 12 months of age is presented, giving basic references data for the investigations of disorders in these regions. These are very common domestic breeds in Brazil. The main indication for diagnostic ultrasound of these digital joints is the evaluation of septic and traumatic disorders of the synovial cavities, the bones and the tendinous structures [17,35].

Easily identifiable anatomic landmarks for the distal limb regions in these young cattle included the joint spaces of the fetlock and the interphalangeal joints. In addition, the bone surfaces such as the characteristic convex shape of the distal metacarpal/metatarsal condyles and the characteristic appearance of fibre bundles with parallel arrangement of the extensor and flexor tendons and the SL served as anatomical landmarks [17,21].

As in studies in adult cattle [17,21,23,24] similar ultrasonographic findings were assessed in these young Nellore and Girlando breeds regarding the appearance of the digital extensor and digital flexor tendons, the bone surfaces and the joint spaces of the interphalangeal and the fetlock joints. The ultrasonographic appearance of the cartilaginous growth plates of the distal metacarpus/metatarsus and the proximal phalanx are imaged as small anechoic interruptions of the hyperechogenic bone surfaces proximal and distal to the joint space of the fetlock joint. They were described in this study for the first time in such a large number of animals. The findings were similar to the growth plates of other long bones in cattle [17,21,27,35].

The transverse plane at the palmar/plantar aspect of the metacarpus/metatarsus about three centimeters proximal to the proximal sesamoidal bones allowed the depiction of the SDFT, the DDFT, the branch of the SL and the SL of the lateral and medial digit in one sonogram. Similar results were reported by other authors [17,23,24,34,36] for adult cattle, however in adult cattle it can be difficult to cover the complete transverse section including both lateral and medial flexor tendons with one sonogram.

In these normal digital joints and digital flexor tendon sheaths of Girolando and Nellore calves the normal synovial fluid content could not be depicted, which is normal. These findings are in accordance with previously reported results in normal distal bovine limbs, where the physiological amount of the synovial fluid within digital joints and the digital flexor tendon sheath could not be imaged [17,21,23,24].

Therefore the cavity of the digital joint pouches and the cavity of the digital flexor tendon sheath surrounding the SDFT, the DDFT and the branch of the SL to the SDFT could not be differentiated in normal cattle. These normal findings make the detection of inflammatory effusions affecting the synovial cavities very easy, because the effusion will create a slight to severe extension of the joint capsules and the tendon sheath wall [17,20,21,35-37].

Even the accurate differentiation of the soft tissue structures in the proximal and the distal part of the digital flexor tendon sheath (pastern region) and the digital joints can act as a helpful prerequisite in patients suffering from disorders of these structures for a subsequent tendovaginoscopic, arthroscopic or other surgical approach [20,37-40].

The ultrasonographic appearance of the DDFT and the SDFT and the ultrasonographic differentiation of the annular ligament in the pastern region have not been described in previous studies [23,24,34]. However, the ultrasonographic and arthroscopic imaging of this annular ligament has been reported recently [37].

Within the study period of four months, a tendency of the dimensions of the SDFT and DDFT to increase over time was appreciated, however without any statistical significance. It can be assumed that the examination period was too short to create significant differences in the dimensions of the flexor tendons. The same chronological limitation can be argued for the sonographic inspection of the cartilaginous growth plates in these calves, it would be interesting to describe their appearance until closure. The square measures of the SDFT, the DDFT and suspensory ligament but unfortunately not the cross-sectional dimensions of these tendons at five different levels of the plamar/plantar metacarpus/metatarsus of Brazilian heifers have been described [34]. In contrast, a strong correlation between the DDFT cross-sectional area and the body weight in horses was noticed by analyzing magnetic resonance images of the distal skeleton of adult horses at different ages and their body weight [40].

The ultrasonographic cross-sectional area of a tendon is considered the most useful and consistent indicator of the normal structure, and quantification (cross-sectional measuremesnts and/or square measures) is routinely accepted on a clinical basis in horses [22,41]. This method has been introduced in the evaluation of the tendons in ruminants [23,24,27]. Studies in horses assume that the cross-sectional area usually increases with tendon fibre injury and in subtle lesions may be the only indication of pathology based on ultrasonography [22,40,42]. Furthermore, comparison between contralateral limbs is considered as a standard clinical procedure in the presence of unilateral clinical evidence of pathology [40,42].

Until now, there has been little investigation of the ultrasonographic appearance of the normal anatomical structures of the distal limbs and the normal dimensions of the digital flexor tendons in calves. The results of this study establish important reference data of the normal structures of the distal limbs (appearance of tendons, joints, bone surfaces, cartilaginous growth plates), and the normal dimensions of the flexor tendons in Nellore and Girolando calves for use in clinical practice. Information on variations in the normal structures, whether through comparison with the contralateral limb or with the same limb of another animal of the same weight and age, could improve our understanding of pathologic processes occurring in the digits of calves.

## Conclusions

Ultrasonography produced consistent images of the normal anatomical structures and measurements of the dimensions of the flexor tendons of the distal limbs in Nellore and Girolando calves.

## Competing interests

The authors declare that they have no competing interests.

## Authors’ contributions

PVRG, NCB and LAFS planned, designed and coordinated the study, performed the experiments and drafted the manuscript; APAC and NB helped perform the experiments; JRC read, analyzed and helped to perform the anatomy study; JK read, analyzed and helped draft the manuscript. All the authors read and approved the final manuscript.
